# FOT Technique Applied for Monitoring of COVID-19 Pneumonia Reveals Small Airways Involvement

**DOI:** 10.3390/diagnostics14111160

**Published:** 2024-05-31

**Authors:** Immanuels Taivans, Laura Grima, Normunds Jurka, Ligita Zvaigzne, Valentina Gordjušina, Gunta Strazda

**Affiliations:** 1 Medical Faculty, University of Latvia, LV1050 Riga, Latvia; grima.lau@gmail.com (L.G.); normunds.jurka@lu.lv (N.J.); valentina.gordjusina@lu.lv (V.G.); gunta.strazda@lu.lv (G.S.); 2 P. Stradiņš University Hospital, LV1002 Riga, Latvia; ligita.zvaigzne@stradini.lv

**Keywords:** forced oscillation technique, small airways, COVID-19 pneumonia

## Abstract

The fact that some SARS-CoV-2 pneumonia patients benefit from changing body position, and some from continuous positive airways pressure (CPAP), indicates the functional character of hypoxia. We hypothesize that such effects could be explained by the closure of small airways. To prove the hypothesis, we evaluated the patency of small airways in 30 oxygen-dependent, spontaneously breathing patients with SARS-CoV-2 pneumonia during their hospital stay using the FOT method and then compared the results with data obtained three months later. During the acute period, total resistance (R5) and peripheral resistance (R5-20) rose above the upper limit of normal (ULN) in 28% and 50% of all patients, respectively. Reactance indices X5, AX and Fres exceeded ULN in 55%, 68% and 66% of cases. Significant correlations were observed between PaO_2_/FiO_2_, the time spent in the hospital and R5, X5, AX and Fres. After 3 months, 18 patients were re-examined. During the hospital stay, 11 of them had risen above the upper limit of normal (ULN), for both resistance (R5-20) and reactance (X5, AX) values. Three months later, ULN for R5-20 was exceeded in only four individuals, but ULN for X5 and AX was exceeded in five individuals. Lung function examination revealed a combined restrictive/obstructive ventilatory failure and reduced CO transfer factor. We interpret these changes as lung tissue remodeling due to the process of fibrosis. We conclude that during acute period of SARS-CoV-2 pneumonia, dilated pulmonary blood vessels and parenchymal oedema induce functional closure of small airways, which in turn induce atelectasis with pulmonary right-to-left shunting, followed by the resulting hypoxemia.

## 1. Introduction

The SARS-CoV-2 infection surprised humanity not only with its rapid spread, but also with a pronounced shortness of breath. The experience gained in treating patients with COVID-19 pneumonia showed that at the basis of hypoxemia lie different mechanisms of pathogenesis, which require specific approaches in treatment [1]. In contrast to the classical SARS infection which manifests with diffuse alveolar damage, SARS-CoV-2 pneumonia proceeds with a mismatch between lung ventilation and perfusion. Using a dual-energy computerized tomography method during the acute period of COVID-19 pneumonia in lungs, both areas with high V/Q ratio and areas with low V/Q ratio can be found [2]. Gattinoni defines lung affections with high V/Q ratio as light or L-type COVID-19 phenotype. These are characterized by high lung compliance, low lung weight and low alveolar recruitment [1]. Camporotta et al. explain reduced perfusion in apparently normal lung areas with high blood plasma angiotensin II level, due to SARS-CoV-2-induced loss of ACE-2 receptors [3]. Also, high-V/Q-ratio areas may develop in lungs in the case of lung vessel thrombosis, which is common for this infection [4].

Low-V/Q-ratio areas are explained by low alveolar ventilation caused by inflammatory process in alveoli or by closure of small airways. In normal lungs, alveolar hypoxia induces local vasoconstriction, but in inflamed lungs this mechanism is suppressed due to accumulation of vasodilating mediators [5]. Vasoplegia that develops in hypoxic lung areas induces venous blood shunting, leading to arterial hypoxemia resistant to oxygen therapy and requiring oxygenation of the extracorporeal membrane (ECMO) [6].

Other methods to improve the oxygenation are aimed at keeping the airways open by applying continuous positive airways pressure (CPAP) and prone positioning of patients [7,8].

Small airways are membranous bronchi with a diameter less than 2 mm. As these airways do not contain cartilaginous rings that oppose compression, they are kept open by the elastic network of the lungs. Elastic forces decrease during the expiration, which induces the collapse of small airways by the end of expiration, especially in the dependent parts of lungs. Other different factors, such as reduced lung elasticity in emphysema, elevated bronchial muscle tone and compression from outside by swollen lung tissue, lead to a low V/Q ratio and pulmonary right-to-left shunting [9,10].

Examination of small airways’ mechanics during the acute period of COVID-19 pneumonia is restricted, because diseased lungs are highly vulnerable and forced manoeuvres used in spirography examinations can induce self-inflicted lung injury (p-SILI) by the patients [11].

Forced oscillation technique (FOT) is a non-invasive method that does not require the patient’s participation and allows to evaluate lung and airways mechanics. The method is based on the application of multifrequent airwaves into patients’ airways during spontaneous breathing and simultaneous recording of changes in the airflow and pressure in the breathing line. This method does not require the cooperation of patients. The oscillations are superimposed on tidal breathing and examination lasts no longer than one minute [12,13]. It allows this method to be used even in patients with severe oxygen deficiency.

Examination of small airways is based on the experimentally established fact that air waves of certain frequencies penetrate airways at a particular depth. For instance, oscillations with frequencies above 20 Hz die before reaching an 8th generation that corresponds to airways with a diameter of 2 mm, while frequencies below 5 Hz reach the lung respiratory zone and are used in studies of lung and respiratory system mechanics. Hence, the range between 5 and 20 Hz is considered as characterizing small airways [14,15,16].

Total impedance to induced air waves consists of resistive, elastic and inertance components. The resistive component is induced by friction of air against the airway walls and depends on the cross-sectional area of the airways. The elastic component, called reactance, reflects the response of the airway wall to the waves induced by the oscillator. This component is delayed by 180 degrees and induces the flow in the breathing line from the opposite direction, and therefore is expressed as a negative ratio of pressure against the flow. Reactance depends on the visco-elastic properties of the airway wall.

The inertance component acts in phase with the resistive component, thus reducing the impact given by elastic recoil. As the inertance grows with increasing oscillation frequencies, both components neutralize each other at the point called the resonance frequency (Fres). Since the inertance component is influenced by air mass and not by the airways, the resonance frequency reflects the properties of the airway wall, similarly to the reactance values [12].

In a multicentre study on 773 asthmatic patients and 99 controls, aimed to assess which tests best measure the presence and extent of small airways disease (SAD) in patients with asthma (ATLANTIS study), it was concluded that those best suited for diagnosis and assessing SAD severity are FOT and spirometry [17].

Based on the above-mentioned observations, we hypothesize that hypoxemia in low-V/Q lung zones may develop due to functional closure of the small airways.

As we could not find any data in the literature on small airways involvement in SARS-CoV-2 pneumonia and its role in pathogenesis of hypoxia, we attempted to study patients during their intrahospital stay using the FOT technique. Data were correlated to severity indices, CT scores and hypoxia indicators. Patients were re-examined three months after the discharge from the hospital and data compared with other lung function characterizing methods.

## 2. Material and Methods

### 2.1. Study Subjects

60 SARS-CoV-2 positive patients treated at P. Stradins University Hospital, Riga, Latvia, were invited to participate in the study between February and May 2021. All patients had signs of pneumonia, confirmed by CT scans. All who gave written consent for participation in the study underwent lung examination with FOT technique. Most patients had various comorbidities besides the COVID-19 infection. Those who required artificial ventilation and those with comorbidities that could influence lung function, like persistent asthma, COPD, or third- and fourth-class heart failure, were not included in the study.

Eventually, 30 COVID-19 pneumonia affected patients were recruited. Among them were patients with type 2 diabetes mellitus and overweight patients. Patients were further classified based on the degree of hypoxemia as mild (200 mm Hg ≤ PaO_2_/FIO_2_ ≤ 300 mm Hg), moderate (100 mm Hg ≤ PaO_2_/FIO_2_ ≤ 200 mm Hg) and severe (PaO_2_/FIO_2_ ≤ 100 mm Hg), according to the Berlin Definition of ARDS severity [13] (Table 1).

As patients were supplied with additional oxygen using different devices, the index FiO_2_ in some cases had to be recalculated from the gas flow rate. In cases when arterial blood samples were not available, PaO_2_ was calculated from SpO_2_ [18].

This study was approved by the Ethics Committee of P. Stradins University hospital. All subjects gave written informed consent.

### 2.2. Study Design

If the patient’s status allowed, FOT measurements were taken every other day of the hospital stay. Other parameters characterizing the patient status, like breathing frequency (BF), SpO_2_ or PaO_2_, SaO_2_ oxygen delivery rate, were recorded as well.

Three months after discharge from the hospital, the patients were invited to repeat FOT measurements and undergo lung function examination, including spirography, body plethysmography and CO transfer test.

### 2.3. Forced Oscillation Technique (FOT)

Lung examination was carried out using the airwave oscillometry system (AOS) (Tremoflo-100 Thorasys, Montreal, QC, Canada). Measurements were performed according to the “Technical standards for respiratory oscillometry”, accepted by ERS official document in 2020 [13]. Since in most cases patients were connected to additional oxygen supply systems via facemasks or nasal cannula, during the examination time, which did not exceed one minute, patients were disconnected from the gas supply system. Measurements were taken in a sitting position using a mouthpiece, containing a bacterial/viral filter, and a nose clip to avoid gas leak. An assistant or the patient himself supported his mouth floor with thumbs and cheeks with remaining fingers to avoid the airwave shunting. Three valid recording trials were performed. Between trials, patient had short rest periods and, if necessary, received oxygen support.

### 2.4. CT Scans

CT scans were usually carried out on the day of admittance to the hospital. In more severe cases, repeated CT scans were performed during the hospital stay. The severity of lung affection was quantitatively assessed via CT scoring [19]. Briefly, each of the five lung lobes was assessed for the degree of involvement and classified as none (0%), minimal (1–25%), mild (26–50%), moderate (51–75%) or severe (76–100%). No involvement corresponded to a lobe score of 0, minimal involvement to a lobe score of 1, mild involvement to a lobe score of 2, moderate involvement to a lobe score of 3 and severe involvement to a lobe score of 4. An overall lung “total severity score” was reached by summing up the five lobe scores (range of possible scores, 0–20) [19].

### 2.5. Analysis

Next, the FOT parameters were checked: respiratory resistance at 5 Hz (R5), indicative of overall respiratory system resistance; frequency dependence of resistance (R5–R20), indicative of small airways obstruction; and three indicators of small airways reactance—resonance frequency (Fres), reactance at 5 Hz (X5) and reactance area (AX)—which characterize the airway wall viscoelasticity. Indices were presented both in absolute values, as a percentage of predicted and as a Z-score, according to reference values from Oostween et al. 2013 [20]. If the Z-score was at a value of 1.645, that was considered to be the upper limit of normal.

Microsoft 365 Excel and Statistca, 6th version were used for statistical data processing.

Before performing the analysis, the conformity of the data to the normal distribution was checked. The distribution was evaluated by Chi-square test and then Kolmogorov–Smirnov test.

Two methods were used for statistical analysis:

(1) a one-way regression analysis, if the dependent and independent factors were continuous.
(2) a one-way repeated measures analysis of variance (repeated ANOVA).

In the case of analysis of variance, the results were expressed as an arithmetic mean ± 95% confidence interval. The difference between the groups was considered significant if *p* < 0.05.

If the distribution did not correspond to normal, a logarithmic or square root transformation was used.

## 3. Results

CT scans revealed ground glass opacifications and reticulation in subpleural areas in the majority of patients. The average CT score, as seen from the Table 1, was around 10 in all severity groups. In rare cases, signs of consolidation or fibrosis were present.

Data in Table 2 suggest that in half of the patients during the acute period of SARS-CoV-2-induced pneumonia, the forced oscillation technique had revealed the increased resistance to airflow in small airways, as indicated by the elevation in the R5-20 index. Two thirds of patients also had elevated reactance indicators AX, X5 and Fres, which is characteristic of high visco-elastance of small airways. Less than one third of patients had elevated total airways resistance R5.

Regression analysis revealed significant associations between FOT values and indicators of the severity of the disease, such as the time spent in the hospital and oxygen dependence. The data are presented in the Table 3.

Oscillometry variables here are presented as the percentages of individually predicted normal levels. The lowest *p*-values, which indicate high association with the oxygenation index (PaO_2_/FiO_2_) and the time spent in hospital, were characteristic for the reactance parameters X5, AX and Fres. Indicator R5-20 did not correlate significantly with indicators of the disease severity. No significant association of FOT indices with CT score was found.

Three months after the discharge from the hospital, 18 patients arrived for repeated control. Using repeated ANOVA method, Table 4 compares the mean changes of oscillometry parameters of the same patients after the period of recovery.

Small airways resistance (R5-20), which during the hospital period was significantly elevated, (317% of predicted value), had returned to the level close to the normal range three months later. At the same time, total resistance (R5), which during the hospital period was only slightly above the predicted values, did not change significantly.

All the reactance values (X5, Fres, AX) which during the hospital period were significantly above the predicted individual levels, had improved significantly three months later, especially AX and Fres, whether expressed as a percentage of predicted values or as a Z-score.

Also, Table 4 shows the values of Z-score and the percentage of patients whose oscillometric parameters were above the upper limit of normal (ULN) during the hospital period and after 3 months. The *p*-value confirms significant reduction of cases with abnormal X5, AX and R5-20 indices after the 3-month recovery period. However, in more than 20% of cases these variables remained elevated, suggesting small airways obstruction in these persons.

Table 5 presents the lung function data of the 18 persons who arrived for repeated control. The most common finding was reduced lung CO transfer factor and low lung volumes -TLC, RV and VC, characteristic of the restrictive pattern of ventilatory failure. The high mean values of FEV1/VC also indicated high restrictive pattern, which was surprising when compared to the high values of reactance indices X5, AX and Fres that are considered indicators of small airways obstruction.

## 4. Discussion

The study has shown that airwave oscillometry performed with a portable device can be successfully applied for monitoring the lung function in oxygen dependent patients with COVID-19 pneumonia. The applied method in patients with a moderate course of disease reveals significant deviations from the normal lung mechanics. The majority of cases could be qualified as heterogenous obstructive pattern with small airways involvement. Observed changes in lung mechanics did not change significantly during the period spent in the hospital but returned to a normal level three months later in the majority of patients.

Indicators of disease severity, such as time spent in the hospital and oxygenation index PaO_2_/FiO_2_, correlated closely with reactance parameters and less closely with resistance oscilllometric parameters. At the same time, no association between CT score and oscillometric indices was found. At first glance the results may look surprising; however, if we take into account that GGO mainly reflects the respiratory lung zone, but oscillometry reflects the conductive zone, this discrepancy does not look so abnormal.

As the frequencies of airwaves used in our study were above 5 Hz, they should not influence the respiratory zone of lungs significantly. Instead, they reflect the changes in the lung conducting zone. Changes in both zones influence the blood oxygenation, but underlying mechanisms in each case are different [21,22].

Processes in the respiratory zone manifest as a diffuse alveolar damage (DAD), directly affects the gas diffusion through alveolo-capillary membrane and can be qualified as organic. Typical pathohistological findings for SARS-CoV-2-induced pneumonia in early disease stage are vascular affections with endotheliolitis, vasculitis with microthrombosis, increased vascular permeability and interstitial oedema. Later, during the proliferation stage, second-type alveolocytes and club cells proliferate and hyaline membranes appear [23,24].

Elevated resistance indicator R5-20 and reactance indices X5, AX and Fres observed in our patients during the hospital stay indicate the closure of significant portion of small airways, that in turn creates low aerated lung zones. The closure developed in dependent lung areas due to small airways compression by lung parenchyma mass elevated by gravity, dilated blood vessels and swollen lung parenchyma [16].

Changes in conducting zone influence the gas supply to alveoli and induce venous blood shunting. The later results from the loss of hypoxic pulmonary vasoconstriction [25].

In this regard, interesting new data about the role of neuron specific enolase (NSE) in SARS-CoV-2 infection have been published. Blood serum analysis for NSE was performed on 323 patients who were referred to hospitals in Catanzaro, Italy. Analysis revealed significantly elevated NSE levels in a cohort of 128 infected patients compared to 178 non-infected patients. NSE levels were markedly higher in the infected patients group with dyspnoea [26]. As NSE is considered as pro-inflammatory mediator [27], it may contribute to vasoplegia and inhibition of hypoxic pulmonary vasoconstriction; this could explain the association between dyspnoea and higher levels of NSE.

Small airways closure observed in acute period in our patients may be qualified as functional, because it changes depending on body position and the phase of breathing. No pathologic changes in small airways are described in pathohistological studies during the acute phase of COVID-19 pneumonia [24,28,29,30].

Airways closure affects the gas exchange between ambient air and the respiratory zone. Independent parts of lung airways may remain closed during the full respiratory cycle. Under normal conditions, local alveolar hypoxia does not lead to hypoxemia because of hypoxic pulmonary vasoconstriction [25]. However, in inflammation, due to the release of local mediators such as nitric oxide, prostaglandins, complement components, bradykinin, etc., vasoplegia develops, which in turn leads to pulmonary right-to-left blood shunting. In SARS-CoV-2 pneumonia, blood shunting may reach severe levels, inducing hypoxemia resistant to oxygen therapy [31].

Using dual-energy CT, it was demonstrated that a considerable fraction of opacifications during the acute period of SARS-CoV-2 pneumonia were hyperperfused despite being diminished or non-ventilated. The authors consider that this finding suggests the presence of a functional intrapulmonary right-to-left shunt [32].

Early airways closure during breathing is a signal of small airways affection. Different detection methods are developed and tested on COVID-19 pneumonia patients. The most widely used in the cases of COVID-19 pneumonia is the quantitative inspiratory-to-expiratory CT matching [33].

During the recovery period after COVID-19, disease quantitative CT analysis of regional ground-glass opacities was performed using inspiratory and expiratory image-matching. Air trapping correlated with the residual volume-to-total lung capacity ratio. Authors found that in survivors of COVID-19, small airways disease was observed even 6 months after the acute period and occurred independently of initial infection severity [33]. Similar studies conducted at different time intervals after the acute period of COVID-19 confirmed the damage to small airways [34,35].

Despite the extensive experience in using this method, it is not suitable for measurements during the acute period of COVID-19 pneumonia, because deep inspiration and full expiration are dangerous manoeuvres that can induce pSILI [11].

For this reason, validation of the FOT method during acute disease stage is problematic. Insight in similar validation gives a large multicenter study of 773 asthmatic patients, comparing them with 99 healthy individuals. The aim of the study was to evaluate the involvement of small airways in cases with a milder or more severe course of the disease. The following methods were compared: spirometry, body plethysmography, impulse oscillometry, multiple breath nitrogen washout and CT (in selected participants). Researchers concluded that impulse oscillometry, which is a version of FOT, and spirometry are most valuable methods for evaluation of small airways involvement in asthma. Authors found no correlation between clinical manifestations of small airways disease (SAD) and CT SAD scores [17].

The functional nature of right-to-left shunting is demonstrated by the experience of oxygen-dependent patients in prone position. The change of the body position from supine to prone redistributes the blood from dorsal to ventral areas of lungs. It opens compressed membranous airways in the dorsal zone of lungs and re-expands collapsed lung units. The effectiveness of this manoeuvre depends on the stage of the disease. It works well in the early disease period that corresponds to high blood shunting. In later stages, characterized by lung tissue consolidation, redistribution does not occur [7,36].

The experience gained in the treatment of COVID-19 patients using CPAP shows that the method is effective only for some patients. As the method is based on keeping airways intraluminal pressure positive to avoid small airways closure, it improves arterial blood oxygenation. The positive effect of CPAP in responders starts immediately, indicating the functional character of small airways closure during the acute period of COVID-19 pneumonia. At the same time, lack of response to CPAP indicates primary affection in the lung respiratory zone [8,37,38].

FOT technique could be applied as a predictor of CPAP effectiveness. It was demonstrated with intrabreath oscillometry, which allows to monitor the changes of resistance (ΔR) and reactance (ΔX) during the course of the respiratory cycle. The application of CPAP at the level of 10 hPa and higher totally abolished any signs of airways closure [39].

Re-examination of patients after 3-month recovery revealed significant improvement of both resistive and reactive components of FOT indices. However, among the returning 18 patients, four still had significantly elevated resistance in small airways (R5-20), and five had reactance indices (X5, AX and Fres) above normal limits. Lung function examination revealed that six patients had a deficient CO transfer factor, five had diminished lung volumes (TLC and RV) and one individual had MMEF below the normal limit. Taken together, the presented values indicated a mixed obstructive/restrictive pattern of ventilatory failure, which is not typical for obstructive lung diseases.

Similar findings have also been reported by Maniscalco et al., who found mixed restrictive/obstructive ventilatory failure in 47% of COVID-19 pneumonia survivors two months after the onset of the disease [40].

Such changes suggest remodeling in lung parenchyma.

Morphological changes in small airways start gradually after two weeks with hyperplasia of club cells followed by their transformation into squamous cells, later invading alveoli and formation of intra-alveolar squamous cell bulbs [24]. Other airways’ epithelial cells undergo molecular alterations typical of epithelial-to-mesenchymal transitions (EMT), which results in the down-regulation of genes associated with tight junctions, and so eradicate the proposed ARDS-protective effect of these epithelial, ACE2-positive cells [41].

Transbronchial biopsies performed 4 to 15 months after the acute period of COVID-19 pneumonia on patients with persistent dyspnoea revealed peribronchial remodeling with interstitial pulmonary fibrosis [42].

Lung mechanics at this phase is characterized by a decrease in lung compliance and increase in pressure swings in oesophageal recordings [43]. The described changes in oscillometric recordings manifest as elevated reactance parameters like X5, AX and Fres, as observed in our study, which reflect increased lung elastance.

It should also be mentioned that FOT instruments that employ frequencies above 5 hz are designed for the diagnosis of airways and therefore do not reflect well the restrictive changes in lung function. FOT devices using lower frequencies were used in early studies of lung mechanics [14,15].

## 5. Conclusions

This study has shown that the FOT method reliably reflects the pathological changes in lung mechanics and allows to monitor the course of COVID-19 disease during both the acute and recovery phase. The study also revealed that typical radiologic and oscillometric findings reflect changes in two distinct lung zones: respiratory and conductive ones.

During the acute phase of disease, closure of small airways is functional and occurs due to compression by swollen lung parenchyma, which leads to hypoxemia due to right-to-left pulmonary blood shunting. After three months of recovery, lung function in the majority of patients returned to the normal range; however, in five persons, a mixed obstructive/restrictive pattern of ventilatory failure was observed, which indicates a persistence of chronic inflammation with lung and airways remodeling. Oscillometric monitoring of COVID-19 pneumonia in the acute period of the disease allows to adjust the CPAP regime and optimize patients’ body positions.

## Figures and Tables

**Table 1:** Patients’ characteristics.

	All Subjects	ARDS			Pneumonia
		Mild	Moderate	Severe	
Subjects	30	3	15	3	9
Age	60.6	28; 64; 75	61 (48–77)	37; 62; 62	47 (33–77)
Sex					
Women	10	1	4	0	5
Men	20	2	11	3	4
BMI	32.3	29.; 37; 38.4	31.3 (23.5–45.8)	30.4; 34.9; 35.4	32.4 (25.5–57.2)
O_2_ budget					
FiO_2_%		36; 44; 44	47 (40–62)	62; 66; 87	26 (21–44)
SpO_2_%		97; 98; 97	95 (90–97)	88; 92; 95	97 (93–100)
PaO_2_ mm Hg (adjusted)		96; 112; 96	77 (40–92)	55; 65; 79	97 (69–145)
PaO_2_/FiO_2_ mmHg		267; 255; 218	151 (115–198)	89; 98; 91	387 (310–536)
CT score		7; 10; 15	10.7 (5–19)	10; 12; 14	10.6 (3–20)

**Table 2:** FOT indices recorded during intrahospital stay in 30 COVID-19 pneumonia patients.

Parameter	Mean	M − 95	M + 95	Z-Score	Z − 95	Z + 95	n > ULN	% > ULN
R5	4.27	3.67	4.87	0.69	0.22	1.16	8/30	27
R5-20	0.95	0.68	1.26	1.72	1.00	2.44	14/30	47
X5	−2.50	−3.09	−1.91	−2.53	−3.58	−1.49	16/30	53
AX	15.78	11.62	21.42	2.15	1.72	2.57	19/30	63
Eres	22.24	19.93	24.82	2.10	1.68	2.52	19/30	63

ULN—upper limit of normal, n > ULN—number of patients exceeding ULN value, % > ULN—percent of patients exceeding ULN value.

**Table 3:** *p*-values of correlation between FOT indices and parameters characterizing COVID-19 pneumonia severity.

Independent Variable on X Axis	Dependent Variable on Y Axis	R Correlation Coefficient	r^2^ (Determination Coefficient)	*p*
PaO_2_/FiO_2_	R5% pred	0.349	0.121	**0.0031**
PaO_2_/FiO_2_	R5-20% pred	−0.173	0.030	0.158
PaO_2_/FiO_2_	X5% pred	0.332	0.110	**0.0051**
PaO_2_/FiO_2_	AX% pred	0.454	0.206	**0.0001**
PaO_2_/FiO_2_	RF% pred	0.434	0.189	**0.0002**
Day before discharge	R5% pred	0.223	0.050	**0.0695**
Day before discharge	R5-20% pred	0.117	0.014	0.348
Day before discharge	RF% pred	0.276	0.076	**0.0237**
Day before discharge	X5% pred	0.409	0.167	**0.0006**
Day before discharge	AX% pred	0.459	0.211	**0.0001**
CT (score)	R5% pred	0.128	0.016	0.445
CT (score)	R5-20% pred	0.050	0.003	0.771
CT (score)	X5% pred	0.233	0.054	0.159
CT (score)	AX% pred	0.242	0.059	0.149
CT (score)	RF% pred	0.198	0.039	0.233

Bold numbers indicate reliability > 95%.

**Table 4:** Patient data during the hospital stay and 3 months after the discharge.

Value	During the Hospital Stay				3 Months after the Discharge				Repeated Measure ANOVA
FOT	Mean	M − 95%	M + 95%	n	Mean	M − 95%	M + 95%	n	*p*
R5	4.34	3.62	5.07	18	3.87	3.11	4.63	18	0.195
R5%	128.2	100.60	155.72	17	109.08	93.91	124.26	17	0.115
R5-20	1.01	0.66	1.43	18	0.62	0.34	0.95	18	**0.0075**
R5-20%	316.7	171.9	583.6	17	122.7	56.5	266.4	17	**0.0048**
X5	−2.53	−3.30	−1.75	18	−2.20	−3.41	−0.99	18	0.619
X5%	202.4	116.1	288.6	17	142.7	68.6	216.8	17	0.225
Fres	22.31	19.01	26.18	18	17.05	14.43	20.15	18	**0.0044**
Fres%	171.9	143.8	200.0	17	130.9	110.9	150.9	17	**0.0081**
AX	17.4	11.1	27.5	18	8.0	4.5	14.4	18	**0.0059**
AX%	448.8	275.5	731.2	17	213.7	131.4	347.6	17	**0.0112**
Z-mean									
R5	0.876	0.152	1.600	18	0.23	−0.10	0.76	18	0.0934
R5-20	2.113	1.025	3.202	18	0.90	0.67	1.72	18	**0.0180**
X5	−3.25	−4.974	−1.522	18	−1.37	−3.11	0.37	18	0.114
AX	2,25	1.581	2.927	18	1.06	0.383	1.75	18	**0.00422**
Fres	1.955	1.286	2.624	17	1.92	0.357	1.44	17	**0.00809**
% > ULN									52,
R5	29.4	12.8	54.2	5	5.9	0.8	32.1	1	0.102
R5-20	64.7	40.4	83.2	11	23.5	9.1	48.6	4	**0.0196**
X5	64.7	40.4	83.2	11	29.4	12.8	54.2	5	**0.0440**
AX	64.7	40.4	83.2	11	29.4	12.8	54.2	5	**0.0440**
Fres	52.9	30.2	74.5	9	29.4	12.8	54.2	5	0.168

FOT—oscillometry variables; Z-mean—mean Z-score values; % > ULN—percent of patients exceeding upper limit of normal; bold fonts indicate *p*-value below 0.05.

**Table 5:** Lung function parameters in COVID-19 pneumonia patients 3 months after discharge from the hospital.

Variable	Mean	M + 95%	M − 95%	Z-Score	Z − 95%	Z + 95%	n	N < −1.65	% < −1.65
VC	3.731	3.317	4.145	−0.138	−0.646	0.371	18	1	5.26
FVC	3.669	3.246	4.092	0.027	−0.458	0.512	18	1	5.26
FEV1	2.905	2.580	3.230	−0.057	−0.461	0.347	18	1	5.26
FEV1/FVC	77.98	75.96	79.99	0.181	−0.144	0.506	18	0	0.00
DLCO	22.19	19.25	25.13	−1.071	−1.511	−0.630	18	6	33.33
KCO	3.938	3.588	4.287	−0.282	−0.654	0.090	18	1	5.56
TLC	5.613	4.852	6.374	−1.284	−1.889	−0.678	14	5	35.71
FRC	2.851	2.392	3.309	−0.943	−1.536	−0.349	14	2	14.29
RV	1.813	1.540	2.085	−1.441	−1.960	−0.923	14	5	35.71
RV/TLC	32.68	28.57	36.79	−1.287	−1.850	−0.724	14	4	28.57
MMEF	2.484	1.963	3.005	−0.679	−1.114	−0.243	14	1	7.14

## Data Availability

Majority of primary data are unavailable because contain private data of patients. For more information please contact the corresponding author.
